# Injectable and biofunctionalized fibrin hydrogels co-embedded with stem cells induce hair follicle genesis

**DOI:** 10.1093/rb/rbac086

**Published:** 2022-10-29

**Authors:** Haiyan Chen, Xiaoxiao Ma, Mengqi Zhang, Zhonghua Liu

**Affiliations:** The National and Local Joint Engineering Laboratory of Animal Peptide Drug Development, College of Life Sciences, Hunan Normal University, Changsha 410081, People’s Republic of China; Tsinghua Shenzhen International Graduate School, Tsinghua University, Shenzhen 518055, People’s Republic of China; The National and Local Joint Engineering Laboratory of Animal Peptide Drug Development, College of Life Sciences, Hunan Normal University, Changsha 410081, People’s Republic of China; The National and Local Joint Engineering Laboratory of Animal Peptide Drug Development, College of Life Sciences, Hunan Normal University, Changsha 410081, People’s Republic of China; The National and Local Joint Engineering Laboratory of Animal Peptide Drug Development, College of Life Sciences, Hunan Normal University, Changsha 410081, People’s Republic of China

**Keywords:** fibrin hydrogels, fibrinogen, hair follicle neogenesis, alopecia, skin-derived precursors

## Abstract

Fibrin-based hydrogels have been widely used in various tissue engineering because of their biocompatibility, biodegradability, tunable mechanical characteristics and nanofibrous structural properties. However, their ability to support stem cells for hair follicle neogenesis is unclear. In this study, we investigated the effect of fibrin hydrogels in supporting skin-derived precursors (SKPs) in hair follicle neogenesis. Our results showed that SKPs in fibrin hydrogels with high cell viability and proliferation, the stemness of SKPs could be maintained, and the expression of hair induction signature genes such as *akp2* and *nestin* was enhanced. Moreover, hair follicle reconstruction experiments showed *de novo* hair genesis in mice and the hairs persisted for a long time without teratoma formation. More importantly, the blood vessels and sebaceous glands were also regenerated. Our study demonstrated that fibrin hydrogels are promising in hair follicle regeneration and have potential application in clinical settings for alopecia and wound healing.

## Graphical Abstract

**Figure:**
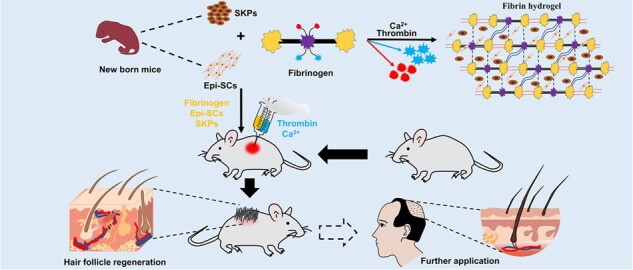

## Introduction

A hair follicle (HF) is an important appendage of the skin, and it is formed based on the signals derived from the dermis during skin morphogenesis [1–3]. Hair is derived from HFs and is involved in thermoregulation, physical protection, sensory activity, social interactions and other important bodily functions [4]. Hair loss (alopecia) is caused by various factors, such as the environment, heredity and pressure, and has become increasingly common worldwide, affecting individuals’ physical, psychological and social well-being [5–7]. The main treatments for hair loss include the oral or local application of drugs, subcutaneous injection therapy, microneedle administration and HF transplantation, but these technologies have limitations and side effects to some extent. FDA-approved treatments for hair loss include oral finasteride and the topical application of minoxidil solution [8]. However, finasteride can cause side effects such as erectile dysfunction and impaired fertility [9]. Owing to the poor solubility of minoxidil in water or other organic solvents that are immiscible with water, ethanol and propylene are often used as solvents, which results in poor skin permeability and can lead to side effects such as skin irritation, scaling and dryness [10]. Considering these adverse consequences, the combination of subcutaneous injection therapy and microneedle administration can solve some problems to a certain extent. Subcutaneous injection therapy includes mesotherapy and platelet-rich plasma (PRP) injection. Mesotherapy is used to directly inject a solution with a variety of nutrients into the subcutaneous HF using an instrument, based on the pressure of water and oxygen, so that the HF can fully absorb nutrients. The current drugs injected with mesotherapy have limitations, and these generally include growth factors, nutrients (such as vitamins), plant extracts (saw palmetto extract) and trace elements, among others, which have little effect on the hair loss caused by HF injury. PRP is rich in a variety of growth factors, such as IGF, epidermal growth factor (EGF) and VEGF, which can effectively promote HF survival [11]. However, recent clinical trial data suggest that PRP might not be effective as a treatment for androgenic alopecia, and in addition, a definitive link between the concentration of growth factors in PRP and the rate of hair growth has not been demonstrated [12]. Moreover, the use of a microneedle combined with minoxidil, growth factors and topical steroid drugs can also be effective for the treatment for hair loss. However, this technology is not very mature, and in a small number of patients treated with microneedles, tension in the heart, inflammation and enlarged lymph nodes, among other negative effects, can be induced [13]. HF transplantation is an effective treatment method that changes the distribution of HFs on the scalp without increasing their number. Its setback is the lack of hair donors, and hair loss is often progressive, leading to various challenges [14].

Unlike other organs that can only be formed in the embryonic stage, HFs can periodically regenerate through interactions between epithelial and dermal stem cells [15]. Based on this, several methods have been used for HF regeneration, including HF stem cell activation through small molecules and drugs, tissue engineering and 3D bioprinting. Seminal studies have reported that papillae isolated from the rat, guinea pig vibrissa and humans could induce HF formation when implanted into the recipient’s non-hairy skin, which indicated that the dermal papilla can reprogram non-hairy epidermis to follicular fate [16–18]. Kageyama *et al*. [19] fabricated collagen-enriched dermal papilla cell aggregates, namely hair beads, which could promote HF regeneration in recipient nude mice. Kang *et al.* [20] bioprinted a multilayer composite structure containing fibroblasts, human umbilical vein endothelial cells, dermal papilla cells and epidermal cells to facilitate regeneration of new tissue-engineered HFs *in vivo*. Skin-derived precursors (SKPs) have been applied for HF regeneration in several studies because of their similarity to dermal papilla cells [18, 21]. Reconstruction of functional HF and sebaceous glands could be achieved through the transplantation of epidermal stem cells (Epi-SCs), SKPs and Matrigel in nude mice [3]. However, above all, HF regeneration relies on stem cells and biomaterials, which are critical factors in tissue engineering.

Scaffolds created from biomaterials are intended to mimic an environment required for stem cells to survive, differentiate and form functional tissue structures [22]. The properties of scaffolds, including stiffness, size, chemical structures, surface area and others will determine the fate of the stem cells. Therefore, it is critical to find suitable scaffold materials for stem cell-induced organogenesis.

Fibrin is a key blood component responsible for hemostasis, and a biopolymer of the monomer fibrinogen has been widely used to engineer various tissues owing to its biocompatibility, biodegradability and tunable mechanical and nanofibrous structural properties [23, 24]. Fibrin, alone or in combination with other materials, has been used as a biological scaffold for stem or primary cells to regenerate adipose tissue, bone, cardiac tissue, cartilage, liver, nervous tissue, ocular tissue, skin, tendons and ligaments [24]. Tan *et al*. [25] applied a fibrin hydrogel co-embedded with bone marrow mesenchymal stem cells and vascular endothelial growth factor to accelerate skin injury repair. It was also reported that fibrin–gelatin hydrogels could comprise excellent biopaper for *in vivo* skin bioprinting [26]. However, whether fibrin hydrogels have potential to support stem cells for HF regeneration is unclear. In this study, fibrin hydrogels were developed based on thrombin and fibrinogen as these can rapidly form a network structure in the presence of calcium ions. The results of this study on the role of fibrin hydrogels in supporting SKPs in HF neogenesis showed that SKPs in fibrin hydrogels had high cell viability and proliferation and that their stemness of SKPs could be maintained. Moreover, the expression of hair-induction signature genes, such as *akp2* and *nestin* was enhanced. Furthermore, HF reconstruction experiments showed *de novo* hair genesis in mice. Our study provides a promising strategy for HF regeneration, with potential applications in the clinical setting of alopecia and wound healing.

## Methods

### Preparation of fibrin hydrogels

Fibrin hydrogels were prepared through Solutions A and B; 100 mg fibrinogen (Yeasen, China) was dissolved in 1 mL 1.8% saline/sodium chloride solution, and 10 mg aprotinin (Sigma, USA) was dissolved in ddH_2_O with the concentration of 170 mg/mL (100×, stock solution), calcium chloride (Sigma, USA) was dissolved in ddH_2_O and the concentration is 42.1 mM (10×, stock solution), 500 U thrombin (sigma, USA) was dissolved in 5 mL 42.1 mM calcium chloride solution (10×, stock solution). The fibrinogen solution and the aprotinin solution was mixed to form Solution A, in which the final concentration of aprotinin is 3.4 mg/mL. The thrombin solution was diluted to 20 U/mL to form Solution B. The Solution A was mixed with Solution B in volume ratio of 1:1 to form fibrin hydrogels at room temperature, the gelation time is approximately 30 s. The cell pellet was suspended with Solution A, which contains 80 mg/mL fibrinogen solution and then mixed with thrombin. The final concentration of fibrinogen is 40 mg/mL, the aprotinin is 1.7 mg/mL and the thrombin is 10U/mL.

### Isolation and culture of Epi-SCs and SKPs

The Epi-SCs and SKPs were isolated from neonatal dorsal skin of C57BL/6 mice 1–3 days after birth as described previously [27, 28]. The dorsal skin was collected and cut into 2–3 mm^2^ slices and digested with 0.3% Dispase II (Sigma, USA) for 60 min at tissue culture incubator. The epidermis and dermis were manually separated and the epidermis was treated with 0.035% collagenase I (Sigma, USA) for 60 min at tissue culture incubator. After digestion, the mixture was filtered with a 70 μm cell strainer, and then centrifuged for 5 min to obtain the Epi-SCs for transplantation. The dermis was treated with 0.35% collagenase I (Sigma, USA) for ∼ 60 min at tissue culture incubator and filtered with a 70 μm cell strainer. The cell suspension was centrifuged and washed with culture medium for two times, cultured in 10-cm non-treated dishes with SKP growth medium and incubated at 37°C in a 5% CO_2_ tissue culture incubator. The SKP growth medium was composed of Dulbecco’s modified Eagle’s medium/F12 (Gibco, USA) in a ratio of 3:1, B27 (Gibco, USA), EGF (Peprotech, USA) and basal fibroblast growth factor (bFGF, Peprotech, USA). The final concentration of EGF is 20 ng/mL and the bFGF is 40 ng/mL.

### Scanning electron microscopy analysis

The interior morphology of fibrin hydrogels was detected by scanning electron microscopy (SEM) and the samples were prepared as previously described [29]. Briefly, the fibrin hydrogels of 20, 40 and 80 mg/mL were quick-frozen in liquid nitrogen, sliced into 100 μm slices and then lyophilized for 72 h by a freeze-drier (LyoQuest-85 PLUS, Telstar, Spain). Subsequently, all the samples were sputter coated with gold and visualized through SEM (Phenom, China).

### Cell proliferation assay

The SKPs proliferation in fibrin hydrogels were evaluated on alarm blue assay (Yeasen, China) following the manufacture’s protocol. Briefly, the SKPs in fibrin hydrogels were cultured in 96-well plates and incubated with 200 μL alarm blue working solution (alarm blue solution: fresh culture medium = 1:10) for 4 h. After incubation, the supernatant solution of all the samples were added to a new 96-well plate and measured the OD value at 570 and 630 nm wavelengths (Epoch2, BioTek, USA). The proliferation rate was calculated and normalized by the OD value on Day 1. The samples were then washed with PBS (Gibco, USA) and replaced with fresh culture medium. Each sample was detected at Days 1, 4 and 7.

### Cell viability

Cell viability of SKPs in fibrin hydrogels was measured through trypan blue stain assay and live/dead staining (KGAF001, KeyGEN BioTECH, China). The SKPs before and cultured in fibrin hydrogels for 3 days were collected and suspended with PBS (Gibco, USA). The cell suspension and 0.4% trypan blue stain solution (Solarbio, China) were mixed at a ratio of 9:1 and the cell viability were automatically calculated in Countstar (Countstar Rigel S2, China). For live/dead staining, the cells were washed with PBS and then incubated with staining solution (PBS: Calcein-AM: PI = 1000:1:1) for 10 min in the dark and then was visualized immediately by a fluorescence microscope (Nikon, Eclipse Ti2-U, Japan).

### Alkaline phosphatase activity

The alkaline phosphatase (AP) activity in SKPs and SKPs encapsulated in fibrin hydrogels was examined on AP staining kit as previously described [30]. Briefly, SKPs and SKPs in fibrin hydrogels were fixed in 4% paraformaldehyde (PFA) for 10 min at room temperature, following washing with PBS. After that, the samples were incubated with 5-bromo-4-chloro-3-indolylphosphate in conjunction with nitro blue tetrazolium (Beyotime Biotechnology, Shanghai, China) solutions at RT for 4 h in the dark. The samples were visualized immediately by a phase-contrast microscope (Nikon, Eclipse Ci-S, Japan).

### Mice for isolating cells and animal experiments

C57BL/6 (7–8 weeks old) and BALB/c nu/nu mice (4–5 weeks old) were purchased from Slac & Jingda Corporation of laboratory animals, Changsha, China. The animals were in a temperature-controlled environment (20°C ± 1°C) and with free access to receive food and water throughout the experiment. This study and all animal procedures were performed with the approval of the Animal Ethics Committee of Hunan Normal University and followed the National Institutes of Health guidelines for the performance of animal experiments.

### Real-time PCR analysis

The relative expression of the samples was detected by real-time PCR (qPCR). Total RNA of the samples was extracted and purified by TRIzol (TAKARA, Japan), and the RNA concentration was quantified by a Nanodrop (ThermoFisher Scientific, USA). The first-strand cDNA was synthesized by the PrimerScriptTM RT Reagent Kit with gDNA Eraser (TAKARA, Japan) and oligo(dT) primers. The qPCR was performed on a SYBR Green Real-Time PCR Mix (TAKARA, Japan) on an analytikJena qTOWER 3G system. The relative expression of target genes was calculated through 2-ΔΔCt method and glyceraldehyde-3-phosphate dehydrogenase (*GAPDH*) was set as an internal control. The primers used were listed in Table 1.

**Table 1.** The primers used for murine gene amplification

Genes	Forward	Reverse
*GAPDH*	CGGAGTCAACGGATTTGGTCGTAT	AGCCTTCTCCATGGTGGTGAAGAC
*Nanog*	TCTTCCTGGTCCCCACAGTTT	GCAAGAATAGTTCTCGGGATGAA
*Oct4*	CACCATCTGTCGCTTCGAGG	AGGGTCTCCGATTTGCATATCT
*c-Myc*	ATGCCCCTCAACGTGAACTTC	CGCAACATAGGATGGAGAGCA
*Sox2*	TCCATGGGCTCTGTGGTCAAG	TGATCATGTCCCGGAGGTCC
*Fibronectin*	ATGTGGACCCCTCCTGATAGT	GCCCAGTGATTTCAGCAAAGG
*a-SMA*	TGAGCAACTTGGACAGCAACA	CTTCTTCCGGGGCTCCTTATC
*Bmp4*	CAGGGAACCGGGCTTGAG	CTGGGATGCTGCTGAGGTTG
*Collagen I*	GCTCCTCTTAGGGGCCACT	CCACGTCTCACCATTGGGG
*Nestin*	GGTTCCCAAAGAGGTGTCCG	CAGCAAACCCATCAGACTCCC
*PDGF-a*	ACGCATGCGGGTGGACTC	GATACCCGGAGCGTGTCAGTTAC
*Akp2*	TCGGAACAACCTGACTGACCC	CTGCTTGGCCTTACCCTCATG

### HF neogenesis

HF reconstruction model was used to evaluate the effect of fibrin hydrogels in supporting stem cells for HF genesis as previous described [30]. Full thickness skin wounds were created on the back of BALB/c nu/nu mice (4–5 weeks old) through skin biopsy punch with diameters of 2, 5 and 10 mm; before that, the mice were anesthetized with sodium pentobarbital (50 mg/kg). The wounds were injected with appropriate volume fibrin hydrogels, which the fibrinogen concentration is 40 mg/mL or Matrigel contained 5 × 10^7^/mL Epi-SCs and 1 × 10^8^/mL SKPs. After transplantation, the mice were covered with Tegaderm (3M, USA) transparent dressing and self-adhering elastic bandage. After transplantation for 4 weeks, some of the mice were sacrificed, the hairs were observed under dissecting microscope (SMZ745, Nikon, Japan) and the wound tissue samples were harvested for histological analysis.

### Immunofluorescence staining

Fresh skin tissue samples were harvested and fixed at 4% PFA overnight, dehydrated through 10, 20 and 30 sucrose gradient for 12 h. The samples were embedded in Tissue Freezing Medium (SAKURA Tissue-Tek^®^ OCT Compound, USA) and stored at –80°C. SKPs samples were fixed at 4% PFA for 30 min and dehydrated in 10, 20 and 30 sucrose gradient for 30 min. The frozen tissue sections of the skin and SKPs samples were incubated with blocking buffer (3% BSA, 10% goat serum) for 2 h at room temperature, and incubated with specific primary antibodies at 4°C overnight. The primary antibodies were listed in Table 2. After incubation, excess primary antibodies were washed with PBS and then incubated with TRITC/cy3 or FITC-conjugated secondary antibody for 2 h at RT. The cell nucleus was stained with 4, 6-diamidino-2-phenylin-dole (DAPI) for 10 min at RT, washed with PBS, and the samples were visualized by confocal microscope (C2, Nikon, Japan).

**Table 2.** Antibody information

Antibody name	Detailed information
Nestin	1:100, ab11306, abcam, UK
Fibronectin	1:100, GTX112794, GeneTex, USA
BMP6	1:100, ab155963, abcam, UK
CD31	1:30, GTX54379, GeneTex, USA
Biotin	1:100, 20Raj1, eBioscience, USA
K 1	1:100, 905601, BioLegend, USA
K 14	1:100, 906004, Biolegend, USA

### Hematoxylin–eosin staining

The regenerated wounds tissue samples were harvested and immediately fixed at 4% PFA for 24 h, dehydrated with 70, 80, 90, 95 and 100% ethanol and then the dehydrated samples were embedded in paraffin. Tissue sections were stained with stained by hematoxylin–eosin (HE) staining kit (Baso, China) as per the instructions. The cell nucleus stained with hematoxylin and the cytoplasm stained with eosin, mounting, and detected with a phase-contrast microscope (Eclipse Ci-S, Nikon, Japan) [27].

### Statistical analysis

All experiments were repeated at least three times, and the results were expressed as mean ± SEM unless stated otherwise. The groups were statistically compared using Student’s t-test and the statistical significance was indicated in each bar, ns: not significant, *P < 0.05, **P < 0.01, ***P < 0.001.

## Results

### Characterization of fibrin hydrogels

**Figure 1.**
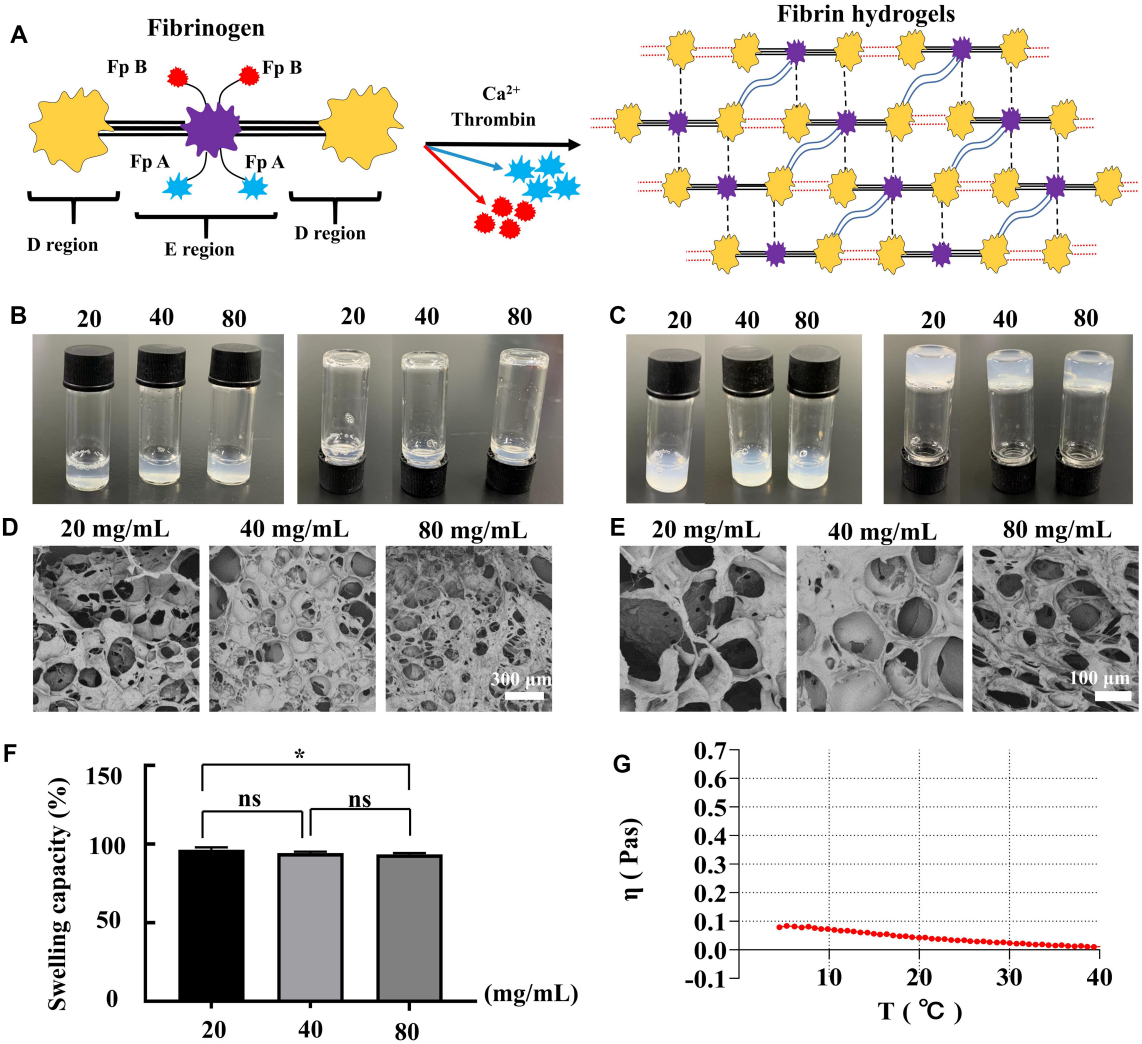
Characterization of fibrin hydrogels. (**A**) The schematic structure of fibrinogen and the fibrin hydrogel formation process. (**B** and **C**) Fibrinogen solution in different concentration and fibrin hydrogel in different concentration. (**D** and **E**) SEM images of different concentration fibrin hydrogels. Scale bar: 300 μm (D), 100 μm (E). (**F**) Swelling capacity of different concentration fibrin hydrogels. (**G**) The viscosity of 80 mg/mL fibrinogen with a change in the temperature.

Figure 1A shows the schematic structure of the fibrinogen and fibrin hydrogel formation process. The fibrinogen solution was transparent, even when the concentration was increased to 80 mg/mL (Fig. 1B), and the fibrinogen solutions at different concentrations immediately formed hydrogels after adding thrombin solution at a 1:1 ratio (Fig. 1C), the gelation time is ∼ 30 s, which is conducive to the subsequent experimental research. SEM results suggested that the fibrin hydrogels had a porous network structure. Moreover, the pore size decreased as the concentration increased (Fig. 1D and E), and the swelling capacity of the fibrin hydrogels also decreased (Fig. 1F). The porous structure is beneficial for nutrient exchange, cell attachment and cell growth, and the cells in fibrin hydrogels have the potential to reproduce the complex structure of native tissues. Materials with higher viscosity are more suitable for providing structural support for tissue scaffolds, whereas materials with lower viscosity are more suitable for cell bioactivity [31]. We detected the viscosity of 80 mg/mL fibrinogen with a change in the temperature, and results showed that the viscosity decreased as the temperature increased. Moreover, the viscosity was relatively low, it was < 0.1 Pa.s at 4°C (Fig. 1G). These results indicated that the solution has good fluidity at room temperature, which is conducive to the uniform mixing of cells. All results indicated that the fibrin hydrogels are beneficial for cell survival.

### Evaluation of SKP compatibility

**Figure 2.**
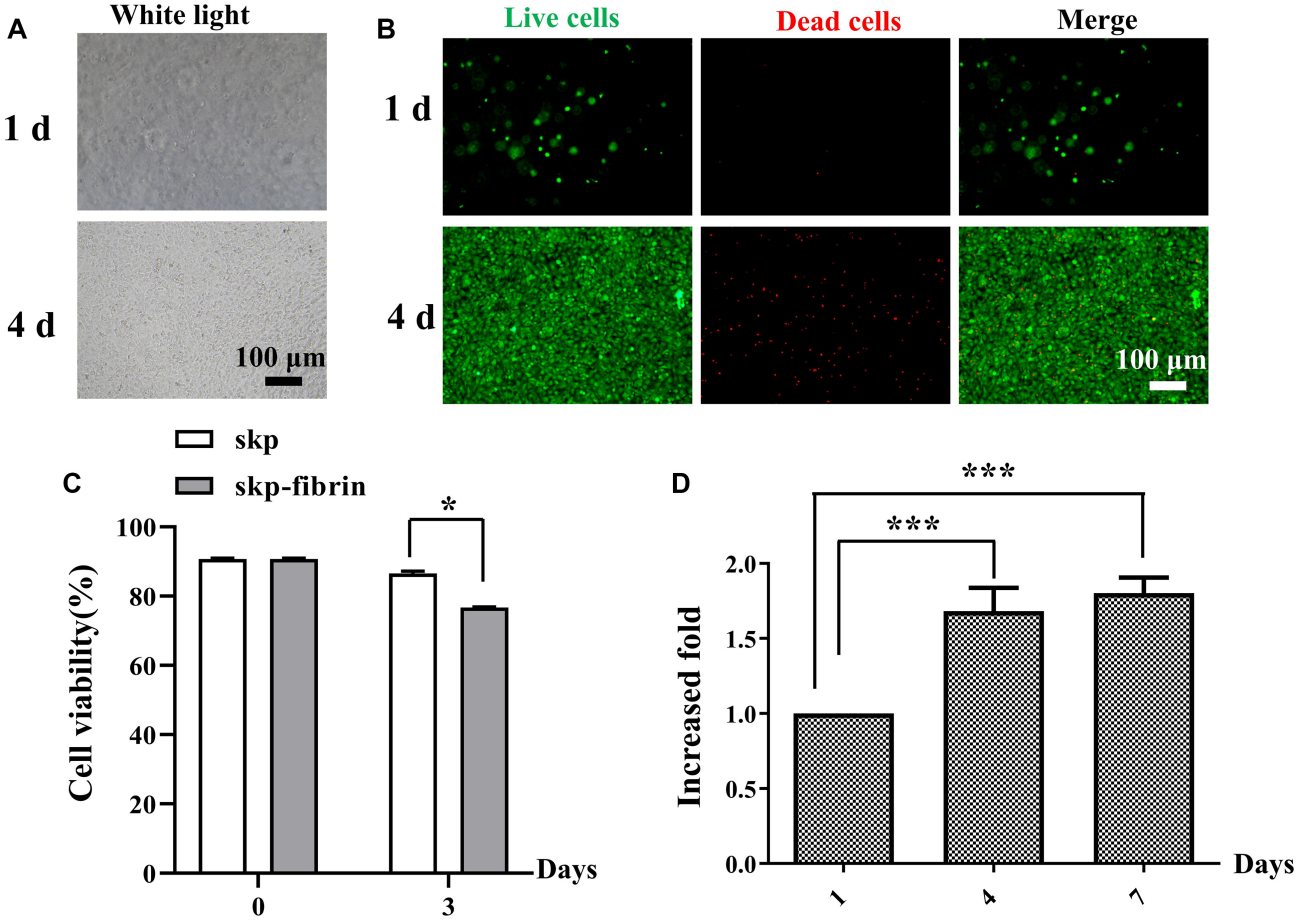
Evaluation of SKPs compatibility. (**A**) Morphology of SKPs in fibrin hydrogels. (**B**) Live and dead staining of SKPs in fibrin hydrogels cultured for 1 day and 4 days, in which live cells are visualized with green and dead cells appear red. Scale bar: 100 μm. (**C**) The viability of SKPs before and after cultured in fibrin hydrogel for 3 days. (**D**) Cell proliferation of SKPs cultured in fibrin hydrogels for 1 day, 4 days and 7 days.

SKPs were co-embedded in fibrin hydrogels and cultured for 7 days to detect cell viability and cell proliferation at different time points. The SKPs in fibrin hydrogels adhered to the hydrogels and spread as the incubation time increased (Fig. 2A and B), indicating that fibrin hydrogels benefit cell attachment. Trypan blue assay results showed that the cell viability of SKPs in fibrin hydrogels was ∼80% (Fig. 2C), which was confirmed by live and dead staining analysis (Fig. 2B). The SKP proliferation was evaluated on Days 1, 4 and 7 using alarm blue assay, and the results showed that the SKPs in fibrin hydrogels could proliferate (Fig. 2D). These results suggested that the fibrin hydrogel scaffolds were able to provide a suitable 3D environment for the survival and growth of SKPs.

### Cytological analysis of SKPs in fibrin hydrogels

**Figure 3.**
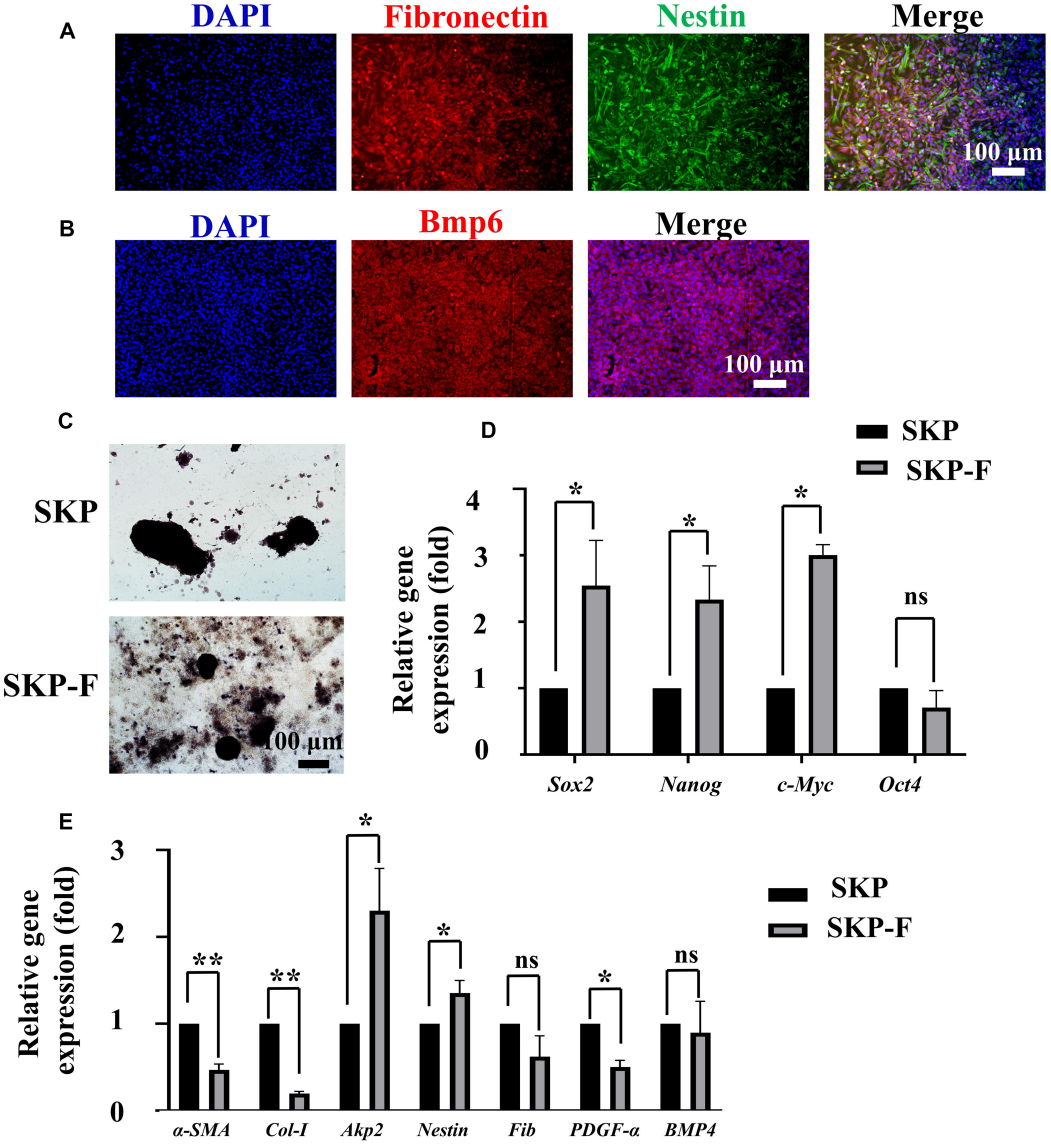
Cytological analysis of SKPs in fibrin hydrogels (SKP-F). (**A** and **B**) Representative immunofluorescence images of BMP6, nestin and fibronectin expression of SKPs in fibrin hydrogels. Scale bar: 100 µm. (**C**) AP staining images of SKPs and SKPs cultured in fibrin hydrogels. Scale bar: 100 µm. (**D** and **E**) Real-time PCR analysis of SKPs in fibrin hydrogels for 3 days for their expression of stemness genes and HF induction-associated genes.

To further evaluate the effect of fibrin hydrogels on SKPs, the SKPs cultured in fibrin hydrogels for 3 days were harvested for immunofluorescence (IF) analysis. From the analysis, it was observed that SKPs in fibrin hydrogels expressed high levels of fibronectin, nestin and BMP6 (Fig. 3A and B), which are typically markers of SKPs. The AP expression level is largely correlated with the hair-inductive ability of DP cells [32]. SKPs have been applied for HF regeneration in many studies owing to their similarity with dermal papilla cells [18, 21]; therefore, we examined the influence of fibrin hydrogels on AP expression by performing an AP staining assay. The staining assay results showed that SKPs in fibrin hydrogels expressed high levels of AP, which were almost the same as those in normal culture (Fig. 3C). Cell stemness is self-renewal and differentiation ability of stem cells, and is a key factor in their clinical application [33]. To evaluate the effect of fibrin hydrogels on SKP stemness and HF induction ability, the pluripotency genes *Oct4*, *Sox2*, *Nanog* and *c-Myc*, and the HF induction-associated genes were detected by qPCR after culturing for 3 days. The results revealed that the expression of the pluripotency genes increased, except for *Oct4* (Fig. 3D), and the expression of HF induction-associated genes *Akp2* and *Nestin* increased, whereas that of *α-SMA, PDGF-α* and C*ol-I* decreased (Fig. 3E). These results suggest that fibrin hydrogels can maintain the properties of SKPs.

### Fibrin hydrogels support stem cell for HF neogenesis

**Figure 4.**
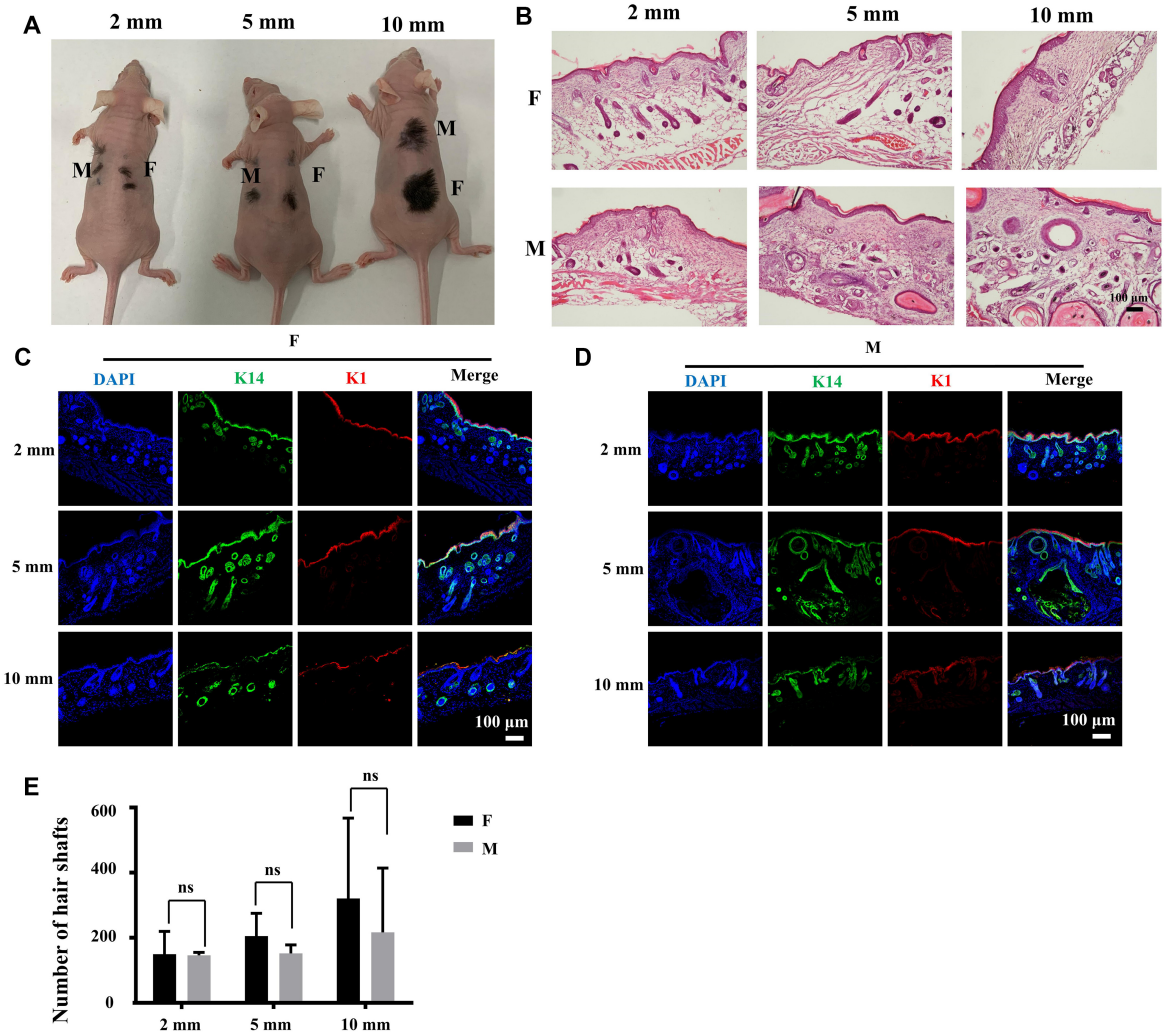
Fibrin hydrogels support stem cell for *de novo* hair genesis. (**A**) Representative images of hair growth observed 4 weeks after transplantation (*N* = 3). (**B**) HE staining of hair genesis tissue shows the hair follicle, epidermis and dermis regenerated. Scale bar: 100 µm. (**C** and **D**) Representative IF images of K1 and K14 in regenerated tissue of fibrin hydrogels and Matrigel. Scale bar: 100 µm. (**E**) The numbers of hair shafts per wound in each wound and each group (*N* ≥ 3).

**Figure 5.**
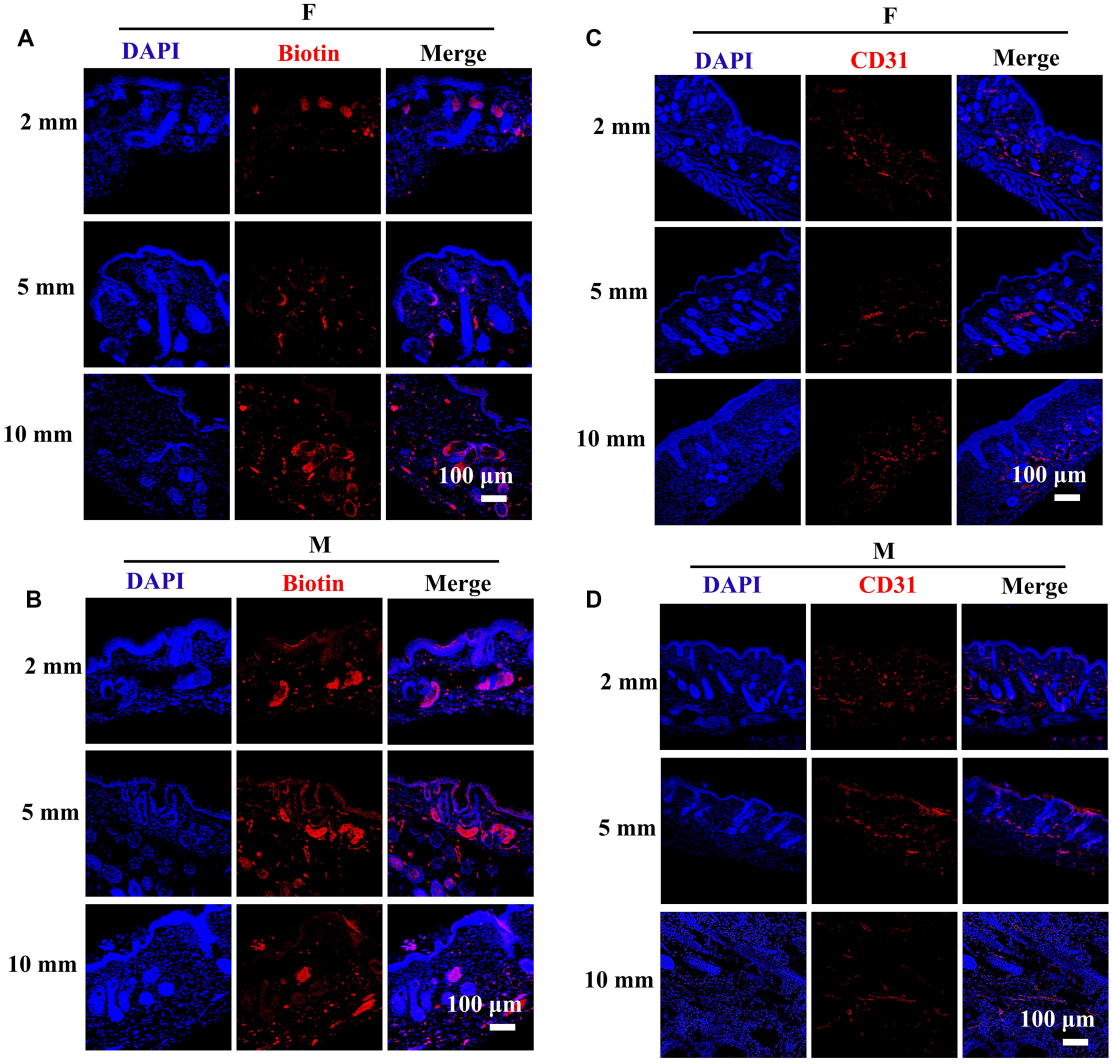
Appendages regenerated after the transplantation of stem cells and fibrin hydrogels. (**A** and **B**) Representative images of IF staining of biotin which showed the regeneration of sebaceous gland in regenerated tissue of fibrin hydrogels and matrigel. Scale bar: 100 µm. (**C** and **D**) Representative images of IF staining of CD31, which showed the regeneration of blood vessels in regenerated tissue of fibrin hydrogels and matrigel. Scale bar: 100 µm.

The fibrin hydrogels were further evaluated through an HF reconstruction model. The Epi-SCs, SKPs and fibrin hydrogels were injected into wounds of 2-, 5- and 10-mm diameters, and stem cells in Matrigel were used as positive controls. After 4 weeks, the newly regenerated hair-inclusive skin tissues in all wounds were imaged under a dissecting microscope, and the relevant hair shafts were counted. The average number of hair shafts increased as the wound area increased in the fibrin hydrogel group, which was similar to that in the Matrigel group (Fig. 4E). Fibrin hydrogels marginally promoted more HF growth relative to that observed for in the Matrigel group, without a significant difference (Fig. 4A and E). HE staining analysis also demonstrated that the HFs regenerated along with the epidermis and dermis (Fig. 4B). Additionally, the blood vessels also regenerated, which was further confirmed through IF staining for CD31, a marker of blood vessels [34] (Fig. 5C and D). Sebaceous glands are other important skin appendages, and thus, we detected the expression of biotin, a specific sebaceous gland marker [3]. IF staining analysis showed that the sebaceous glands regenerated in the wounds (Fig. 5A and B). It has been reported that keratin 1 (K1) is expressed in differentiated keratinocytes and that keratin 14 (K14) is expressed in Epi-SCs [35, 36]; therefore, the HFs were further evaluated by performing IF staining for K1 and K14. The results showed that the regenerated tissue contained the epidermis, dermis and HFs, and the epidermis was stratified, which was similar to the natural skin (Fig. 4C and D). These results indicated that fibrin hydrogels are suitable for *de novo* HF regeneration and wound healing.

### Biocompatibility evaluation

**Figure 6.**
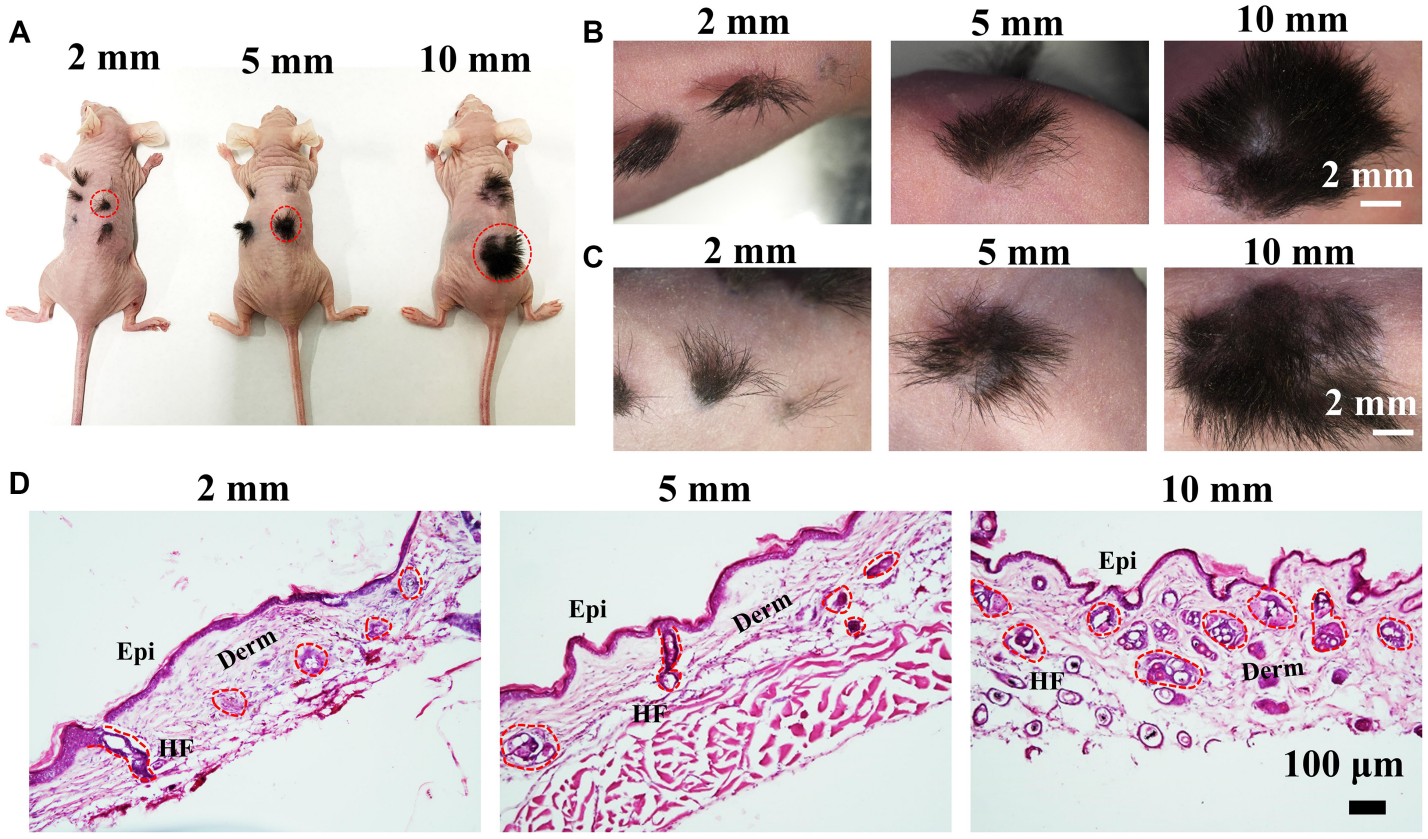
Biocompatibility evaluation. (**A**) The morphological images of mice after transplantation of 6 months. (**B** and **C**) Representative images of the hair genesis tissue after transplantation of 4 weeks and 6 months. Scale bar: 2 mm. (**D**) HE staining images of regenerated tissue after transplantation for 6 months. Scale bar: 100 µm.

The biocompatibility of biomedical materials must be considered and evaluated when they are applied. To detect the durability of neogenic black hair shafts and the skin stem cell biocompatibility with fibrin hydrogels, some mice were observed for 6 months after transplantation. Morphological images showed that the mice still presented with the growth of thick hair even after 6 months (Fig. 6A–C). HE staining analysis was applied to further evaluate the regenerated tissue, and the results showed that the HFs remained, although with some hair loss, and there was no teratoma formation (Fig. 6D). These results suggest that fibrin hydrogels are suitable for HF regeneration and wound healing in clinical settings.

## Discussion

An increasing number of people, especially at a young age, are suffering from hair loss, which seriously affects their physical and mental health. There are several treatments for hair loss; among them, stem cell-based tissue engineering and regenerative medicine are becoming the most thriving approach for the treatment of hair loss, aiming to reconstruct functional HFs to replace or repair damaged or lost HFs [18]. Previous studies have shown that many stem cell-based tissue engineering techniques have achieved hair regeneration at the laboratory stage. For example, pluripotent stem cells from adipose [37], bone marrow [38], HF [39] and umbilical cord blood [40] multipotent stem cell transplantation can regenerate HFs in the skin. However, due to the limitations of these cells, such as tumorigenicity and infection transmission, tight regulations, short shelf life, and strict production, transport and storage conditions, their widespread application has been limited [41]. DP cells are widely used to study hair regeneration. The induction of HFs by DP cells originates from embryonic development but is not limited to embryonic developmental stages, and DP cells from postnatal skin can still promote dermal sheath cells and non-follicle-associated fibroblasts during skin remodeling and wound healing [42]. Nevertheless, the number of DP cells is very small, their availability is limited, and it is difficult to maintain their HF-inductive ability *in vitro* [43]. SKPs are pluripotent stem cells extracted from rodent and human skin, and have been shown to share the same niche as DP cells, as reported in previous studies [21]. It is well known that DP cells are essential for the induction of HF regeneration, and these functional properties of SKPs suggest their potential application in the biogenesis of skin substitutes for regenerating HFs [44]. In this study, we selected SKPs as seed cells for tissue engineering.

Biological scaffolds have always been the focus of regenerative medicine based on tissue engineering, in addition to seed cell selection. In recent years, many researchers have selected various biological scaffolds for HF regeneration. Dong *et al*. [45] selected the silk fibroin–sodium alginate scaffold to study its influence on the induction ability of HF cells, proving that this composite scaffold had a good biomimetic extracellular matrix (ECM) structure, which could maintain the morphological shape of dermal papilla cells and aggregate growth characteristics. However, this composite scaffold did not regenerate blood vessels. Zhang *et al*. [46] constructed a composite dressing with a polylactic acid fiber membrane and zinc–silicon bioceramics, which could effectively activate HF cells to participate in the re-epithelialization and blood vessel formation of burned skin. However, since the liquid in this dressing is stored in the ceramic powder in the middle layer, it can only be transferred in one direction [46]. Although other substances such as collagen–chitosan scaffolds and leucine-activated nanohybrid biofilms had been proven to promote HF regeneration, they all had certain limitations [47, 48]. Based on the catalytic effect of thrombin on fibrinogen, thrombin and fibrinogen were used as raw materials to form a three-dimensional network structure with good biocompatibility under the action of calcium ions. Fibrin is an ECM protein, which plays a crucial role in the coagulation cascade and is a scaffold for tissue repair after injury. It is formed by thrombin and CaCl_2_ polymerized fibrinogen, resulting in the formation of three-dimensional network of fibrin fibers [49, 50]. Fibrin-based hydrogels proliferate, migrate and differentiate into specific tissues or organs via ECM secretion, promoting efficient seeding and uniform distribution of cells, and resorbing gradually due to the action of proteases [51, 52]. The gelation time of fibrin hydrogels can be changed according to the special requirements based on the composition and the concentration of the components. Taking advantage of these physiological properties and cell–material interactions, fibrin matrices have been widely applied in tissue engineering and have been approved for biomedical use in the USA [53–55]. The fibrin hydrogels in this study could be formed in ∼ 30 s at room temperature, which is conducive to the subsequent experimental research and increases the possibility of clinical application.

This study found that fibrin hydrogels with Epi-SCs and SKPs have a satisfactory effect in supporting HF neogenesis. We transplanted the SKPs into the fibrin hydrogel, and the SKPs adhered well to the hydrogel and survived and proliferated, which was related to the porous network structure of fibrinogen. Furthermore, SKPs in fibrin hydrogels maintained the expression of SKP markers Bmp6, fibronectin and nestin, and genes such as *Akp2* and *nestin*, which are involved in the HF-inducing properties of DP cells. At the same time, in our study, the vascular molecular marker CD31, sebaceous gland marker biotin and HF marker keratin were expressed in regenerated wound tissue. These results suggested that fibrin hydrogels can support HFs and other regenerated appendages. The most prominent finding is that we observed partially transplanted mice for 6 months, and they still showed thick hair growth; this shows that the fibrin hydrogel has extremely good biocompatibility. The question of whether the regenerated HFs could be sustained for a long duration requires more time and additional detection, which is also a key factor for clinical applications. To realize the rapid proliferation of Epi-SCs and SKPs from humans or to find new types of cells for human HF regeneration, further study is needed. In conclusion, this study demonstrated that fibrin hydrogel with Epi-SCs and SKPs is a promising method for HF regeneration in a clinical setting.

## Conclusion

Whether fibrin hydrogels could be applied for HF neogenesis was determined through *in vitro* experiments and *in vivo* HF reconstruction experiments. We demonstrated that SKPs in fibrin hydrogels have high cell viability and proliferation, their stemness could be maintained, and the expression of hair induction signature genes, such as *akp2* and *nestin*, was enhanced. Moreover, HF reconstruction experiments showed *de novo* hair genesis in mice, and components such as sebaceous glands and blood vessels were also regenerated. Interestingly, the regenerated hairs could persist for a long time without teratoma formation. With further advances, the fibrin hydrogels with Epi-SCs and SKPs comprise a potential therapeutic approach for alopecia and wound healing.

## Funding

This research was supported by National Natural Science Foundation of China (32000956) and the Science and Technology Innovation Program of Hunan Province (2020RC4023).

*Conflicts of interest statement*. The authors declare that they have no conflict of interest.

## Supplementary Material

rbac086_Supplementary_Data

## References

[1] Wang X , Tredget EE, Wu Y. Dynamic signals for hair follicle development and regeneration. Stem Cells Dev 2012;21:7–18. 21787229 10.1089/scd.2011.0230

[2] Hunt DP , Morris PN, Sterling J, Anderson JA, Joannides A, Jahoda C, Compston A, Chandran S. A highly enriched niche of precursor cells with neuronal and glial potential within the hair follicle dermal papilla of adult skin. Stem Cells 2008;26:163–72. 17901404 10.1634/stemcells.2007-0281

[3] Wang X , Wang X, Liu J, Cai T, Guo L, Wang S, Wang J, Cao Y, Ge J, Jiang Y, Tredget EE, Cao M, Wu Y. Hair follicle and sebaceous gland De novo regeneration with cultured epidermal stem cells and skin-derived precursors. Stem Cells Transl Med 2016;5:1695–706. 27458264 10.5966/sctm.2015-0397 PMC5189649

[4] Schneider MR , Schmidt-Ullrich R, Paus R. The hair follicle as a dynamic miniorgan. Curr Biol 2009;19:R132–42. 19211055 10.1016/j.cub.2008.12.005

[5] Hadshiew IM , Foitzik K, Arck PC, Paus R. Burden of hair loss: stress and the underestimated psychosocial impact of telogen effluvium and androgenetic alopecia. J Invest Dermatol 2004;123:455–7. 15304082 10.1111/j.0022-202X.2004.23237.x

[6] Marks DH , Naftulin JS, Penzi LR, Manatis-Lornell A, Yasuda MR, Chapman CM, Rao SR, Saavedra A, Senna MM. Histologic and clinical cross-sectional study of chronic hair loss in patients with cutaneous chronic graft-versus-host disease. J Am Acad Dermatol 2019;81:1134–41. 31454500 10.1016/j.jaad.2019.03.031

[7] Marks DH , Penzi LR, Ibler E, Manatis-Lornell A, Hagigeorges D, Yasuda M, Drake LA, Senna MM. The medical and psychosocial associations of alopecia: recognizing hair loss as more than a cosmetic concern. Am J Clin Dermatol 2019;20:195–200. 30390206 10.1007/s40257-018-0405-2

[8] Price VH , Menefee E, Strauss PC. Changes in hair weight and hair count in men with androgenetic alopecia, after application of 5% and 2% topical minoxidil, placebo, or no treatment. J Am Acad Dermatol 1999;41:717–21. 10534633 10.1016/s0190-9622(99)70006-x

[9] Shin YS , Karna KK, Choi BR, Park JK. Finasteride and erectile dysfunction in patients with benign prostatic hyperplasia or male androgenetic alopecia. World J Mens Health 2019;37:157–65. 30209896 10.5534/wjmh.180029 PMC6479090

[10] Suchonwanit P , Thammarucha S, Leerunyakul K. Minoxidil and its use in hair disorders: a review. Drug Des Devel Ther 2019;13:2777–86. 10.2147/DDDT.S214907 PMC6691938 31496654

[11] Pavlovic V , Ciric M, Jovanovic V, Stojanovic P. Platelet rich plasma: a short overview of certain bioactive components. Open Med (Wars) 2016;11:242–7. 28352802 10.1515/med-2016-0048 PMC5329835

[12] Abdin R , Zhang Y, Jimenez JJ. Treatment of androgenetic alopecia using PRP to target dysregulated mechanisms and pathways. Front Med (Lausanne) 2022;9:843127. 35372424 10.3389/fmed.2022.843127 PMC8965895

[13] Kumar MK , Inamadar AC, Palit A. A randomized controlled, Single-Observer blinded study to determine the efficacy of topical minoxidil plus microneedling versus topical minoxidil alone in the treatment of androgenetic alopecia. J Cutan Aesthet Surg 2018;11:211–6. 30886475 10.4103/JCAS.JCAS_130_17 PMC6371730

[14] Rose PT. Hair restoration surgery: challenges and solutions. Clin Cosmet Investig Dermatol 2015;8:361–70. 10.2147/CCID.S53980 PMC4507484 26203266

[15] Sennett R , Rendl M. Mesenchymal-epithelial interactions during hair follicle morphogenesis and cycling. Semin Cell Dev Biol 2012;23:917–27. 22960356 10.1016/j.semcdb.2012.08.011 PMC3496047

[16] Horne KA , Jahoda CA, Oliver RF. Whisker growth induced by implantation of cultured vibrissa dermal papilla cells in the adult rat. J Embryol Exp Morphol 1986;97:111–24. 3794596

[17] Jahoda CA , Oliver RF, Reynolds AJ, Forrester JC, Horne KA. Human hair follicle regeneration following amputation and grafting into the nude mouse. J Invest Dermatol 1996;107:804–7. 8941664 10.1111/1523-1747.ep12330565

[18] Castro AR , Logarinho E. Tissue engineering strategies for human hair follicle regeneration: how far from a hairy goal? Stem Cells Transl Med 2020;9:342–50. 31876379 10.1002/sctm.19-0301 PMC7031632

[19] Kageyama T , Yan L, Shimizu A, Maruo S, Fukuda J. Preparation of hair beads and hair follicle germs for regenerative medicine. Biomaterials 2019;212:55–63. 31103946 10.1016/j.biomaterials.2019.05.003

[20] Kang D , Liu Z, Qian C, Huang J, Zhou Y, Mao X, Qu Q, Liu B, Wang J, Hu Z, Miao Y 3. Bioprinting of a gelatin-alginate hydrogel for tissue-engineered hair follicle regeneration. Acta Biomater 2022. 10.1016/j.actbio.2022.03.011 35288311

[21] Fernandes KJ , McKenzie IA, Mill P, Smith KM, Akhavan M, Barnabe-Heider F, Biernaskie J, Junek A, Kobayashi NR, Toma JG, Kaplan DR, Labosky PA, Rafuse V, Hui CC, Miller FD. A dermal niche for multipotent adult skin-derived precursor cells. Nat Cell Biol 2004;6:1082–93. 15517002 10.1038/ncb1181

[22] Wang X , Wang J, Guo L, Wang X, Chen H, Wang X, Liu J, Tredget EE, Wu Y. Self-assembling peptide hydrogel scaffolds support stem cell-based hair follicle regeneration. Nanomedicine 2016;12:2115–25. 27288668 10.1016/j.nano.2016.05.021

[23] de Melo BAG , Jodat YA, Cruz EM, Benincasa JC, Shin SR, Porcionatto MA. Strategies to use fibrinogen as bioink for 3D bioprinting fibrin-based soft and hard tissues. Acta Biomater 2020;117:60–76. 32949823 10.1016/j.actbio.2020.09.024

[24] Ahmed TA , Dare EV, Hincke M. Fibrin: a versatile scaffold for tissue engineering applications. Tissue Eng Part B Rev 2008;14:199–215. 18544016 10.1089/ten.teb.2007.0435

[25] Tan J , Li L, Wang H, Wei L, Gao X, Zeng Z, Liu S, Fan Y, Liu T, Chen J. Biofunctionalized fibrin gel co-embedded with BMSCs and VEGF for accelerating skin injury repair. Mater Sci Eng C Mater Biol Appl 2021;121:111749. 33579437 10.1016/j.msec.2020.111749

[26] Hakam MS , Imani R, Abolfathi N, Fakhrzadeh H, Sharifi AM. Evaluation of fibrin-gelatin hydrogel as biopaper for application in skin bioprinting: an in-vitro study. Biomed Mater Eng 2016;27:669–82. 28234249 10.3233/BME-161617

[27] Chen H , Wang X, Chen Y, Han J, Kong D, Zhu M, Fu X, Wu Y. Pten loss in Lgr5(+) hair follicle stem cells promotes SCC development. Theranostics 2019;9:8321–31. 31754399 10.7150/thno.35467 PMC6857063

[28] Wang X , Dong S, Wu Y. Isolation and cultivation of epidermal (stem) cells. Methods Mol Biol 2019;1879:133–8. 29582374 10.1007/7651_2018_132

[29] Chen H , Liang L, Lin Z, Zhang Y, Mi S, Rao L, Xu T. 3D bioprinted cancer cells are more tolerant to serum starvation than 2D cells due to autophagy. Mater Today Chem 2022;24:100912.

[30] Zhao W , Chen H, Zhang Y, Zhou D, Liang L, Liu B, Xu T. Adaptive multi-degree-of-freedom in situ bioprinting robot for hair-follicle-inclusive skin repair: a preliminary study conducted in mice. Bioeng Transl Med 2022;7:e10303. 36176617 10.1002/btm2.10303 PMC9472011

[31] Bhattacharya M , Malinen MM, Lauren P, Lou YR, Kuisma SW, Kanninen L, Lille M, Corlu A, GuGuen-Guillouzo C, Ikkala O, Laukkanen A, Urtti A, Yliperttula M. Nanofibrillar cellulose hydrogel promotes three-dimensional liver cell culture. J Control Release 2012;164:291–8. 22776290 10.1016/j.jconrel.2012.06.039

[32] Rendl M , Polak L, Fuchs E. BMP signaling in dermal papilla cells is required for their hair follicle-inductive properties. Genes Dev 2008;22:543–57. 18281466 10.1101/gad.1614408 PMC2238674

[33] Reisman M , Adams KT. Stem cell therapy: a look at current research, regulations, and remaining hurdles. P T 2014;39:846–57. 25516694 PMC4264671

[34] Liu L , Shi GP. CD31: beyond a marker for endothelial cells. Cardiovasc Res 2012;94:3–5. 22379038 10.1093/cvr/cvs108

[35] Gonzales KAU , Fuchs E. Skin and its regenerative powers: an alliance between stem cells and their niche. Dev Cell 2017;43:387–401. 29161590 10.1016/j.devcel.2017.10.001 PMC5797699

[36] Lamb R , Ambler CA. Keratinocytes propagated in serum-free, feeder-free culture conditions fail to form stratified epidermis in a reconstituted skin model. PLoS One 2013;8:e52494. 23326335 10.1371/journal.pone.0052494 PMC3543440

[37] Wu J , Yang Q, Wu S, Yuan R, Zhao X, Li Y, Wu W, Zhu N. Adipose-derived stem cell exosomes promoted hair regeneration. Tissue Eng Regen Med 2021;18:685–91. 34173219 10.1007/s13770-021-00347-y PMC8325725

[38] Park J , Jun EK, Son D, Hong W, Jang J, Yun W, Yoon BS, Song G, Kim IY, You S. Overexpression of Nanog in amniotic fluid-derived mesenchymal stem cells accelerates dermal papilla cell activity and promotes hair follicle regeneration. Exp Mol Med 2019;51:1–15. 10.1038/s12276-019-0266-7 PMC6802618 31273189

[39] Gao X , Wang Q, Yuan L, Jiao C, Yu Y, Wang X, Xu P, Ma Y, Wu Y, Wu Z, Li L, Xiao J, Dang Y. REGgamma regulates hair cycle by activating Lgr5 positive hair follicle stem cells. J Dermatol Sci 2021;102:101–8. 33933312 10.1016/j.jdermsci.2021.04.002

[40] Bak DH , Choi MJ, Kim SR, Lee BC, Kim JM, Jeon ES, Oh W, Lim ES, Park BC, Kim MJ, Na J, Kim BJ. Human umbilical cord blood mesenchymal stem cells engineered to overexpress growth factors accelerate outcomes in hair growth. Korean J Physiol Pharmacol 2018;22:555–66. 30181702 10.4196/kjpp.2018.22.5.555 PMC6115345

[41] Yuan AR , Bian Q, Gao JQ. Current advances in stem cell-based therapies for hair regeneration. Eur J Pharmacol 2020;881:173197. 32439260 10.1016/j.ejphar.2020.173197

[42] Driskell RR , Clavel C, Rendl M, Watt FM. Hair follicle dermal papilla cells at a glance. J Cell Sci 2011;124:1179–82. 21444748 10.1242/jcs.082446 PMC3115771

[43] Wang J , Wang X, Xie J, Yao B, Mo M, Ma D, Huang C, Xu R, Fu X, Tredget EE, Wu Y. Engineered skin substitute regenerates the skin with hair follicle formation. Biomedicines 2021;9:400. 33917746 10.3390/biomedicines9040400 PMC8068101

[44] Biernaskie J , Paris M, Morozova O, Fagan BM, Marra M, Pevny L, Miller FD. SKPs derive from hair follicle precursors and exhibit properties of adult dermal stem cells. Cell Stem Cell 2009;5:610–23. 19951689 10.1016/j.stem.2009.10.019 PMC2828150

[45] Dong K , Wang X, Shen Y, Wang Y, Li B, Cai C, Shen L, Guo Y. Maintaining inducibility of dermal follicle cells on silk fibroin/sodium alginate scaffold for enhanced hair follicle regeneration. Biology (Basel) 2021;10:269. 33810528 10.3390/biology10040269 PMC8066588

[46] Zhang Z , Li W, Liu Y, Yang Z, Ma L, Zhuang H, Wang E, Wu C, Huan Z, Guo F, Chang J. Design of a biofluid-absorbing bioactive sandwich-structured Zn-Si bioceramic composite wound dressing for hair follicle regeneration and skin burn wound healing. Bioact Mater 2021;6:1910–20. 33364530 10.1016/j.bioactmat.2020.12.006 PMC7750441

[47] Xia Y , Chen J, Ding J, Zhang J, Chen H. IGF1- and BM-MSC-incorporating collagen-chitosan scaffolds promote wound healing and hair follicle regeneration. Am J Transl Res 2020;12:6264–76. 33194028 PMC7653568

[48] Lin X , Li Y, Luo W, Xiao L, Zhang Z, Zhao J, Liu C, Li Y. Leucine-activated nanohybrid biofilm for skin regeneration via improving cell affinity and neovascularization capacity. J Mater Chem B 2020;8:7966–76. 32756660 10.1039/d0tb00958j

[49] Ross JJ , Tranquillo RT. ECM gene expression correlates with in vitro tissue growth and development in fibrin gel remodeled by neonatal smooth muscle cells. Matrix Biol 2003;22:477–90. 14667840 10.1016/s0945-053x(03)00078-7

[50] Willerth SM , Arendas KJ, Gottlieb DI, Sakiyama-Elbert SE. Optimization of fibrin scaffolds for differentiation of murine embryonic stem cells into neural lineage cells. Biomaterials 2006;27:5990–6003. 16919326 10.1016/j.biomaterials.2006.07.036 PMC1794024

[51] Park CH , Woo KM. Fibrin-based biomaterial applications in tissue engineering and regenerative medicine. Adv Exp Med Biol 2018;1064:253–61. 30471038 10.1007/978-981-13-0445-3_16

[52] Geer DJ , Swartz DD, Andreadis ST. Fibrin promotes migration in a three-dimensional in vitro model of wound regeneration. Tissue Eng 2002;8:787–98. 12459057 10.1089/10763270260424141

[53] Sproul E , Nandi S, Brown A. Fibrin biomaterials for tissue regeneration and repair. In: Barbosa MA, Cristina M, Martins L (eds). Peptides and Proteins as Biomaterials for Tissue Regeneration and Repair. Woodhead Publishing, 2018, pp. 151–73.

[54] Mol A , van Lieshout MI, Dam-de Veen CG, Neuenschwander S, Hoerstrup SP, Baaijens FP, Bouten CV. Fibrin as a cell carrier in cardiovascular tissue engineering applications. Biomaterials 2005;26:3113–21. 15603806 10.1016/j.biomaterials.2004.08.007

[55] Yang C , Chung N, Song C, Youm HW, Lee K, Lee JR. Promotion of angiogenesis toward transplanted ovaries using nitric oxide releasing nanoparticles in fibrin hydrogel. Biofabrication 2022;14:011001. 10.1088/1758-5090/ac3f28 34852328

