# Supplementary material for: Injectable and biofunctionalized fibrin hydrogels co-embedded with stem cells induce hair follicle genesis
Source: Regen Biomater. 2022 Oct 29;10:rbac086. doi: 10.1093/rb/rbac086 (PMC9847531; doi:10.1093/rb/rbac086)
Supplement: rbac086_Supplementary_Data [file rbac086_supplementary_data.docx]

**Point-by-point response to the reviewers’ comments**

Sep 25^th^, 2022

Dear editor and dear Reviewers,

Thank you for giving us the opportunity to submit a revised draft of the manuscript titled "**Injectable and biofunctionalized fibrin hydrogels co-embedded with stem cells induce hair follicle genesis**". We appreciate the time and effort that you and the reviewers have dedicated to providing your constructive comments on our manuscript. We have been able to incorporate changes to reflect the insightful suggestions provided by the reviewers. A detailed point-by-point response to the reviewers’ comments is listed below. New text passages in the manuscript are written in blue color.

We are very grateful for all your comments and for publication of this paper. If you have any questions, we shall be at your disposal.

Sincerely,

Haiyan Chen, PhD

Development of the National and Local Joint Engineering Laboratory of Animal Peptide Drug,

College of Life Sciences,

Hunan Normal University,

Changsha, 410081, China

TEL: +86 15973166956

E-mail: chy654221336@hunnu.edu.cn

**Comments from** **Reviewer #1**

Reviewer #1: The quality of data and manuscript seems to be improved.

**Reviewer #1-1.** Comments to the Author

There are some spacing mistakes in manuscript including figure legends so you need to fix it for enhanced reading for readers.

**Response to Reviewer #1-1.**

Thanks for your suggestion. We have revised our manuscript include figure legends carefully, and the changes were marked in red in the revised manuscript.

**Reviewer #1-2.** In an introduction section, you said, “The main treatments for hair loss include drug therapy and HF transplantation. HF transplantation is an effective treatment method that changes the distribution of HFs on the scalp without increasing their number. The setback is the lack of hair donors, and hair loss is often progressive, leading to various challenges [8].” You need to add more alopecia treatment platform in recent years and justify your study’s necessity. For instance, you can suggest current hair loss treatment platform’s problem and safety issue and emphasize your platform’s strength by comparing with existing treatment such as oral, topical, hypodermic injection, hair transplantation, or microneedle.

**Response to Reviewer #1-2.**

We appreciate the reviewer’s constructive suggestion. We have revised our introduction section according to your suggestions as follows.

**Page 3, lines 49–78:** “*The main treatments for hair loss include the oral or local application of drugs, subcutaneous injection therapy, microneedle administration, and HF transplantation, but these technologies have limitations and side effects to some extent. FDA-approved treatments for hair loss include oral finasteride and the topical application of minoxidil solution [8]. However, finasteride can cause side effects such as erectile dysfunction and impaired fertility [9]. Owing to the poor solubility of minoxidil in water or other organic solvents that are immiscible with water, ethanol and propylene are often used as solvents, which results in poor skin permeability and can lead to side effects such as skin irritation, scaling and dryness [10]. Considering these adverse consequences, the combination of subcutaneous injection therapy and microneedle administration can solve some problems to a certain extent. Subcutaneous injection therapy includes mesotherapy and platelet-rich plasma (PRP) injection. Mesotherapy is used to directly inject a solution with a variety of nutrients into the subcutaneous HF using an instrument, based on the pressure of water and oxygen, so that the HF can fully absorb nutrients. The current drugs injected with mesotherapy have limitations, and these generally include growth factors, nutrients (such as vitamins), plant extracts (saw palmetto extract), and trace elements, among others, which have little effect on the hair loss caused by HF injury. PRP is rich in a variety of growth factors, such as IGF, EGF and VEGF, which can effectively promote HF survival [11]. However, recent clinical trial data suggest that PRP might not be effective as a treatment for androgenic alopecia, and in addition, a definitive link between the concentration of growth factors in PRP and the rate of hair growth has not been demonstrated [12]. Moreover, the use of a microneedle combined with minoxidil, growth factors and topical steroid drugs can also be effective for the treatment for hair loss. However, this technology is not advanced, and in a small number of patients treated with microneedles, tension in his heart, inflammation and enlarged lymph nodes, among other negative effects, can be induced [13]. HF transplantation is an effective treatment method that changes the distribution of HFs on the scalp without increasing their number. Its setback is the lack of hair donors, and hair loss is often progressive, leading to various challenges [14].”*

**Reviewer #1-3.** There is no explanation of Figure 3D & 3E, the values in y-axis had broken boxes. You need to enhance your image quality.

**Response to Reviewer #1-3.**

Thanks for your advice. We have revised our Figure 3D & 3E (Figure 1-2) to improve the image quality, and added the explanations as follows.

**Page 14, lines 308-314:** *“To evaluate the effect of fibrin hydrogels on SKP stemness, the pluripotency genes Oct4, Sox2, Nanog, and c-Myc and the HF induction-associated were detected by real-time PCR after culturing for 3 days. The results revealed that the expression of the pluripotency genes increased, except for Oct4 (Figure 3D), and the expression of HF induction-associated genes akp2 and nestin increased, whereas that of α-SMA and col-II decreased (Figure 3E). These results suggest that fibrin hydrogels can maintain the properties of SKPs.”*

**Page 12, lines 263-269:** *“(D, E) Real time PCR analysis of SKPs in fibrin hydrogels for 3 days for their expression of stemness genes and HF induction-associated genes”.*

*Figure 1(Figure 3D) Real time PCR analysis of SKPs in fibrin hydrogels for 3 days for their expression of stemness genes.*

*Figure 2 (Figure 3E) Real time PCR analysis of SKPs in fibrin hydrogels for 3 days for their expression of HF induction-associated genes.*

**Reviewer #1-4.** In figure 1D, the porous structure was detected and the pore size decreased following the concentration. You need to discuss this phenomenon’s meaning on your research. You need to show data this difference of pore size can cause.

**Response to Reviewer #1-4.**

Thank you for your advice. We have revised our manuscript and discussed this phenomenons according to your advice. A major advantage of hydrogel as a matrix for tissue engineering is that it has a similar structure to human extracellular matrix and can covalently bind with peptide ligands of cell membrane receptors, thus stimulating the adhesion, diffusion, and growth of cells in the gel matrix [1]. The porous network structure of hydrogels facilitates the transport of nutrients and the inward growth of cells, and the difference in pore size is mainly due to the differences in the degree of cross-linking of the hydrogels [2]. SEM results suggested that the fibrin hydrogels had a porous network structure and the pore size decreased as the concentration increased, and the swelling capacity of the fibrin hydrogels also decreased (Figure 3). The results indicated that the pore size affect the swelling capacity of the fibrin hydrogels. We have also added discussion in the results section as follows.

**Page 11, line 256-261:** “*SEM results suggested that the fibrin hydrogels had a porous network structure. Moreover, the pore size decreased as the concentration increased (Figure 1D-E), and the swelling capacity of the fibrin hydrogels also decreased (Figure 1F). The porous structure is beneficial for nutrient exchange, cell attachment, and cell growth, and the cells in fibrin hydrogels have the potential to reproduce the complex structure of native tissues.”*

The SEM experiments here were intended to demonstrated the fibrin hydrogels had a porous network and this structure is beneficial for nutrient exchange, cell attachment, cell growth and proliferation. Cultured cells in a microenvironment with appropriate pore size can relatively control the three-dimensional spatial organization and reproduce the complex structure of native tissues more accurately.

Figure 3 (Figure 1F) Swelling capacity of different concentration fibrin hydrogels.

**References:**

[1]. M.M. Hasani-Sadrabadi, P. Sarrion, S. Pouraghaei, Y. Chau, S. Ansari, S. Li, T. Aghaloo, A. Moshaverinia, An engineered cell-laden adhesive hydrogel promotes craniofacial bone tissue regeneration in rats, Sci Transl Med 12(534) (2020).

[2]. L. Wei, J. Tan, L. Li, H. Wang, S. Liu, J. Chen, Y. Weng, T. Liu, Chitosan/Alginate Hydrogel Dressing Loaded FGF/VE-Cadherin to Accelerate Full-Thickness Skin Regeneration and More Normal Skin Repairs, Int J Mol Sci 23(3) (2022).

**Reviewer #1-5.** In figure 6D, your staining image shows a loss of tissue and the hair follicles’ phase seems that it is still on telogen phase. Even though you transplanted your hair follicles into the skin, it may be hard to sustain the implanted follicles.

**Response to Reviewer #1-5.**

Thank you for your comment. There is really a loss of tissue in Figure 6D in the process of HE staining, we have revised our figures (Figure 4) and we hope it can reach the requirements now. The hair follicles in Figure 6D are indeed on telogen phase, it was from the tissue which was harvested 6 months after transplantation. From Figure 6D, we could observe hair follicles (marked in red lines), although some hair follicles are not completed which were caused by the process of making tissue sections and the regenerated hair follicles do not have the directionality of normal hair follicles. Figure 6D shows that the density of regenerated hair follicles is very similar to that of normal hair follicles, and is basically the same as that of normal hair follicles (Figure 5) [1]. Appearance of mice 6 months after transplantation shows some hair loss, maybe some hair follicles also loss, but most hair follicles are sustained. It may be hard to sustain the implanted hair follicles, whether the regenerated HFs could be sustained for a long duration requires more time and further study. We have also discussed this issue in the discussion section as follows.

**Page 24, line 446-451:** “*The most prominent finding is that we observed partially transplanted mice for six months, and they still showed thick hair growth (Figure 6); this shows that the fibrin hydrogel has extremely good biocompatibility. The question of whether the regenerated HFs could be sustained for a long duration requires more time and additional detection, which is also a key factor for clinical applications.”*

Figure 4 (Figure 6D) HE staining images of regenerated tissue after transplantation for 6 months. Scale bar: 100 µm. Epi: epidermis, Derm: dermis, HF: hair follicle.

Figure 5 HE staining images of normal skin from different age mice. Scale bar: 50 µm. Epi: epidermis, Derm: dermis, HF: hair follicle [1].

**References:**

[1]. Chen H, Wang X, Han J, Fan Z, Sadia S, Zhang R, Guo Y, Jiang Y, Wu Y: AKT and its related molecular feature in aged mice skin. PLoS One 2017, 12(6):e0178969.

**Reviewer #1-6.** In discussion section, you need to strengthen your discussion following your figure legends. It is hard to read because there is no direction with figure number on your discussion.

**Response to** **Reviewer #1-6.**

Thank you for your suggestion. We have revised our discussion according to your suggestions as follows.

**Page 22, line 384-454: *“****An increasing number of people, especially at a young age, are suffering from hair loss, which seriously affects their physical and mental health. There are several treatments for hair loss; among them, stem cell-based tissue engineering and regenerative medicine are becoming the most thriving approach for the treatment of hair loss, aiming to reconstruct functional HFs to replace or repair damaged or lost HFs [18]. Previous studies have shown that many stem cell-based tissue engineering techniques have achieved hair regeneration at the laboratory stage. For example, pluripotent stem cells from adipose [36], bone marrow [37], HF [38], and umbilical cord blood [39] multipotent stem cell transplantation can regenerate HFs in the skin. However, due to the limitations of these cells, such as tumorigenicity and infection transmission, tight regulations, short shelf life, and strict production, transport, and storage conditions, their widespread application has been limited [40]. DP cells are widely used to study hair regeneration. The induction of HFs by DP cells originates from embryonic development but is not limited to embryonic developmental stages, and DP cells from postnatal skin can still promote dermal sheath cells and non-follicle-associated fibroblasts during skin remodeling and wound healing [41]. Nevertheless, the number of DP cells is very small, their availability is limited, and it is difficult to maintain their HF-inductive ability in vitro [42]. SKPs are pluripotent stem cells extracted from rodent and human skin, and have been shown to share the same niche as DP cells, as reported in previous studies [21]. It is well known that DP cells are essential for the induction of HF regeneration, and these functional properties of SKPs suggest their potential application in the biogenesis of skin substitutes for regenerating HFs [43]. In this study, we selected SKPs as seed cells for tissue engineering.*

*Biological scaffolds have always been the focus of regenerative medicine based on tissue engineering, in addition to seed cell selection. In recent years, many researchers have selected various biological scaffolds for HF regeneration. Dong et al. selected the silk fibroin-sodium alginate scaffold to study its influence on the induction ability of HF cells, proving that this composite scaffold had a good biomimetic extracellular matrix structure, which could maintain the morphological shape of dermal papilla cells and aggregate growth characteristics. However, this composite scaffold did not regenerate blood vessels [44]. Zhang et al. constructed a composite dressing with a polylactic acid fiber membrane and zinc-silicon bioceramics, which could effectively activate HF cells to participate in the re-epithelialization and blood vessel formation of burned skin. However, since the liquid in this dressing is stored in the ceramic powder in the middle layer, it can only be transferred in one direction [45]. Although other substances such as collagen-chitosan scaffolds and leucine-activated nanohybrid biofilms had been proven to promote HF regeneration, they all had certain limitations [46, 47]. Based on the catalytic effect of thrombin on fibrinogen, thrombin and fibrinogen were used as raw materials to form a three-dimensional network structure with good biocompatibility under the action of calcium ions. Fibrin is an extracellular matrix (ECM) protein, which plays a crucial role in the coagulation cascade and is a scaffold for tissue repair after injury. It is formed by thrombin and CaCl2 polymerized fibrinogen, resulting in the formation of three-dimensional network of fibrin fibers [48, 49]. Fibrin-based hydrogels proliferate, migrate and differentiate into specific tissues or organs via ECM secretion, promoting efficient seeding and uniform distribution of cells, and resorbing gradually due to the action of proteases [50, 51].The gelation time of fibrin hydrogels can be changed according to the special requirements based on the composition and the concentration of the components Taking advantage of these physiological properties and cell-material interactions, fibrin matrices have been widely applied in tissue engineering and have been approved for biomedical use in the United States [52-54]. The fibrin hydrogels in this study could be formed in approximately 30 s at room temperature (Figure 1B-C), which is conducive to the subsequent experimental research and increases the possibility of clinical application.*

*This study found that fibrin hydrogels with Epi-SCs and SKPs have a satisfactory effect in supporting HF neogenesis. We transplanted the SKPs into the fibrin hydrogel, and the SKPs adhered well to the hydrogel and survived and proliferated, which was related to the porous network structure of fibrinogen. Furthermore, SKPs in fibrin hydrogels maintained the expression of SKP markers Bmp6, fibronectin, and nestin, and genes such as Akp2 and nestin, which are involved in the HF-inducing properties of DP cells (Figures 2-3). At the same time, in our study, the vascular molecular marker CD31, sebaceous gland marker biotin, and HF marker keratin were expressed in regenerated wound tissue (Figures 4-5). These results suggested that fibrin hydrogels can support HFs and other regenerated appendages. The most prominent finding is that we observed partially transplanted mice for six months, and they still showed thick hair growth (Figure 6); this shows that the fibrin hydrogel has extremely good biocompatibility. The question of whether the regenerated HFs could be sustained for a long duration requires more time and additional detection, which is also a key factor for clinical applications. To realize the rapid proliferation of Epi-SCs and SKPs from humans or to find new types of cells for human HF regeneration, further study is needed. In conclusion, this study demonstrated that fibrin hydrogel with Epi-SCs and SKPs is a promising method for HF regeneration in a clinical setting.”*

**Reviewer #2:**

Comments to the Author

This article reports a kind of fibrin hydrogels that can effectively regenerate hair follicles, which have been mainly demonstrated by *in vitro* and *in vivo* biological studies. It is an interesting work for tissue regeneration of hair follicles that have useful application. While major concerns can be found below and should be reconsidered and revised before for possible publication.

**Reviewer #2-1.** The preparation of fibrin hydrogel is not clear, e.g., temperature, Ca2+ concentration, and the gelation time that is very important. So In results and discussion, these points need to be discussed briefly for readers.

**Response to Reviewer #2-1.**

Thank you for your constructive advice. We have revised the manuscript and added the details including temperature, Ca^2+^ concentration, and these points have been discussed in the results and discussion as follows.

**Page 6, line 125-135:** ***“****Fibrin hydrogels were prepared through solution A and solution B. 100 mg fibrinogen (Yeasen, China) was dissolved in 1 mL 1.8% saline/sodium chloride solution, and 10 mg aprotinin (sigma, USA) was dissolved in ddH_2_O with the concentration of 170 mg/mL (100×, stock solution), calcium chloride (Sigma, USA) was dissolved in ddH_2_O and the concentration is 42.1 mM (10×, stock solution), 500 U thrombin (sigma, USA) was dissolved in 5 mL 42.1 mM calcium chloride solution (10×, stock solution). The fibrinogen solution and the aprotinin solution was mixed to form solution A, in which the final concentration of aprotinin is 3.4 mg/mL.”*

**Page 11, line 252-256:** *“The fibrinogen solution was transparent, even when the concentration was increased to 80 mg/mL (Figure 1B), and the fibrinogen solutions at different concentrations immediately formed hydrogels after adding thrombin solution at a 1:1 ratio (Figure 1C), the gelation time is approximately 30s, which is conducive to the subsequent experimental research*.*”*

**Page 23, line 425-436:** *“It is formed by thrombin and CaCl_2_ polymerized fibrinogen, resulting in the formation of three-dimensional network of fibrin fibers [48, 49]. Fibrin-based hydrogels proliferate, migrate and differentiate into specific tissues or organs via ECM secretion, promoting efficient seeding and uniform distribution of cells, and resorbing gradually due to the action of proteases [50, 51].The gelation time of fibrin hydrogels can be changed according to the special requirements based on the composition and the concentration of the components Taking advantage of these physiological properties and cell-material interactions, fibrin matrices have been widely applied in tissue engineering and have been approved for biomedical use in the United States [52-54]. The fibrin hydrogels in this study could be formed in approximately 30 s at room temperature (Figure 1B-C), which is conducive to the subsequent experimental research and increases the possibility of clinical application.”*

**References:**

[1] B. Tan, S. Kuang, X. Li, X. Cheng, W. Duan, J. Zhang, W. Liu, Y. Fan, Stereotactic technology for 3D bioprinting: from the perspective of robot mechanism, Biofabrication 13(4) (2021).

**Reviewer #2-2.** As scaffold, the mechanical properties are very important for tissue regeneration, this point is lacking, which needs to be studied and discussed in main text.

**Response to** **Reviewer #2-2.**

Thank you for valuable suggestion. We have revised and discussed mechanical properties in the main text. Viscosity reflects the flow resistance of an object. Materials with higher viscosity are more suitable for providing structural support for tissue scaffolds, while materials with lower viscosity are more suitable for cell bioactivity [1]. Firstly, we have detected the viscosity of 80 mg/mL fibrinogen with the change of temperature, and the viscosity results showed that the viscosity was decreased with the temperature increased, and the viscosity was relatively small, it was less than 0.1 Pas at 4 °C (Figure 6) .These results indicated that the solution has good fluidity at room temperature, which is conducive to the uniform mixing of cells. At the same time, fibrin hydrogel is rapidly gelatinized, which is conducive to rapid wound coverage and reduces the loss of cells and hydrogels.

A major advantage of hydrogel as a matrix for tissue engineering is that it has a similar structure to human extracellular matrix and can covalently bind with peptide ligands of cell membrane receptors, thus stimulating the adhesion, diffusion, and growth of cells in the hydrogels [2]. The porous network structure of hydrogels facilitates the transport of nutrients and the inward growth of cells, and the difference in pore size is mainly due to the differences in the degree of cross-linking of the hydrogels [3]. So we have examined the structure of fibrin hydrogels on SEM, and also detected the swelling capacity of the fibrin hydrogels. SEM results suggested that the fibrin hydrogels had a porous network structure and the pore size decreased as the concentration increased (Figure 7), and the swelling capacity of the fibrin hydrogels also decreased (Figure 8). This results also suggested the fibrin hydrogels had a porous network and this structure is beneficial for nutrient exchange, cell attachment, cell growth and proliferation, and the cells in fibrin hydrogels has the possibility to reproduce the complex structure of native tissues.

Fibrin is a key blood component responsible for hemostasis, and a biopolymer of the monomer fibrinogen, which has been widely used for engineering various tissues owing to its biocompatibility, biodegradability, and tunable mechanical and nanofibrous structural properties [4-5]. Moreover, fibrin hydrogels have commercial product. For example, in clinical applications, commercial fibrin glue (FG) is used to prevent anastomotic leakage during pancreaticojejunostomy, fibrin glue spray is used to treat cases of diffuse bleeding, and it is used as an adhesive during skin graft surgery, enabling early initiation of related physiotherapy [6-7]. All the above indicated that fibrin is a kind of high biological safety and has high clinical application value. In addition to this, fibrin, alone or in combination with other materials, has been used as a biological scaffold for stem or primary cells to regenerate adipose tissue, bone, cardiac tissue, cartilage, liver, nervous tissue, ocular tissue, skin, tendons, and ligaments [5]. Tan *et al*. applied a fibrin hydrogel co-embedded with bone marrow mesenchymal stem cells and vascular endothelial growth factor to accelerate skin injury repair [8]. It was also reported that fibrin-gelatin hydrogels could be excellent biopaper for *in vivo* skin bioprinting [9]. All these suggested that fibrin hydrogels are suitable for clinical applications, the mechanical properties of fibrin hydrogels could meet the clinical application through the addition of other components or change the concentration of the components and so on. We have added these results and discussion in the revised manuscript as follows.

Figure 6 (Figure 1G) The viscosity of 80 mg/mL fibrinogen with a change in the temperature.

Figure 7 (Figure 1D-E) SEM images of different concentration fibrin hydrogels. Scale bar: 300 μm (D), 100 μm (E).

Figure 8 (Figure 1F) Swelling capacity of different concentration fibrin hydrogels.

**Page 11, line 256-261:** “*SEM results suggested that the fibrin hydrogels had a porous network structure. Moreover, the pore size decreased as the concentration increased (Figure 1D-E), and the swelling capacity of the fibrin hydrogels also decreased (Figure 1F). The porous structure is beneficial for nutrient exchange, cell attachment, and cell growth, and the cells in fibrin hydrogels have the potential to reproduce the complex structure of native tissues.”*

**Page 11, line 261-268:** *“Materials with higher viscosity are more suitable for providing structural support for tissue scaffolds, whereas materials with lower viscosity are more suitable for cell bioactivity^30^. We detected the viscosity of 80 mg/mL fibrinogen with a change in the temperature, and results showed that the viscosity decreased as the temperature increased. Moreover, the viscosity was relatively low, it was less than 0.1 Pas at 4 °C. These results indicated that the solution has good fluidity at room temperature, which is conducive to the uniform mixing of cells. All results indicated that the fibrin hydrogels are beneficial for cell survival.”*

**Page 5, line 102-110: *“****Fibrin is a key blood component responsible for hemostasis, and a biopolymer of the monomer fibrinogen has been widely used to engineer various tissues owing to its biocompatibility, biodegradability, and tunable mechanical and nanofibrous structural properties [23, 24]. Fibrin, alone or in combination with other materials, has been used as a biological scaffold for stem or primary cells to regenerate adipose tissue, bone, cardiac tissue, cartilage, liver, nervous tissue, ocular tissue, skin, tendons, and ligaments [24]. Tan et al. applied a fibrin hydrogel co-embedded with bone marrow mesenchymal stem cells and vascular endothelial growth factor to accelerate skin injury repair [25]. It was also reported that fibrin-gelatin hydrogels could comprise excellent biopaper for in vivo skin bioprinting [26].”*

**References:**

[1]. Bhattacharya, M. et al. Nanofibrillar cellulose hydrogel promotes three-dimensional liver cell culture. J Control Release 164, 291-298, doi:10.1016/j.jconrel.2012.06.039 (2012).

[2]. M.M. Hasani-Sadrabadi, P. Sarrion, S. Pouraghaei, Y. Chau, S. Ansari, S. Li, T. Aghaloo, A. Moshaverinia, An engineered cell-laden adhesive hydrogel promotes craniofacial bone tissue regeneration in rats, Sci Transl Med 12(534) (2020).

[3]. L. Wei, J. Tan, L. Li, H. Wang, S. Liu, J. Chen, Y. Weng, T. Liu, Chitosan/Alginate Hydrogel Dressing Loaded FGF/VE-Cadherin to Accelerate Full-Thickness Skin Regeneration and More Normal Skin Repairs, Int J Mol Sci 23(3) (2022).

[4]. de Melo, B. A. G. et al. Strategies to use fibrinogen as bioink for 3D bioprinting fibrin-based soft and hard tissues. Acta Biomater 117, 60-76, doi:10.1016/j.actbio.2020.09.024 (2020).

[5]. Ahmed, T. A., Dare, E. V. & Hincke, M. Fibrin: a versatile scaffold for tissue engineering applications. Tissue Eng Part B Rev 14, 199-215, doi:10.1089/ten.teb.2007.0435 (2008).

[6] J.W. Baker, W.D. Spotnitz, S.P. Nolan, A technique for spray application of fibrin glue during cardiac operations, Ann Thorac Surg 43(5) (1987) 564-5.

[7] S. Tashiro, E. Murata, T. Hiraoka, K. Nakakuma, E. Watanabe, Y. Miyauchi, New technique for pancreaticojejunostomy using a biological adhesive, Br J Surg 74(5) (1987) 392-4.

[8]. Tan, J. et al. Biofunctionalized fibrin gel co-embedded with BMSCs and VEGF for accelerating skin injury repair. Mater Sci Eng C Mater Biol Appl 121, 111749, doi:10.1016/j.msec.2020.111749 (2021).

[9]. Hakam, M. S., Imani, R., Abolfathi, N., Fakhrzadeh, H. & Sharifi, A. M. Evaluation of fibrin-gelatin hydrogel as biopaper for application in skin bioprinting: An in-vitro study. Biomed Mater Eng 27, 669-682, doi:10.3233/BME-161617 (2016).

**Reviewer #2-3.** Hair follicles studies: as commercial Fibrin glue is more similar to the fribrin hydrogels in this paper, why the authors have not used it as control.

**Response to Reviewer #2-3.**

Thank you for pointing this out. What your mentioned is a good idea. In this study, we are also intended to find out a hydrogel for hair follicle regeneration which has the potential to be applied in clinical. The specific composition and concentration of the commercial fibrin glue were not available, so we have not used it as control in this study. We firstly detected whether fibrin hydrogels in this study could support stem cells for *de novo* hair follicle regeneration, and our results demonstrated that the fibrin hydrogels could support SKP survival and regeneration, and support *de novo* hair follicle regeneration. In this study, we chose Matrigel as positive control which has used in a myriad of cell-culture applications for more than four decades due to it could closely mimic the natural extracellular matrix secreted by adherent cells [1-2], and our previous work demonstrated that Matrigel have the ability of supporting *de novo* hair follicle regeneration [3]. In the upcoming trials, we will conduct preclinical trials and detect the commercial fibrin hydrogels, and hope one day these could be really applied in clinical.

[1] Reed J, Walczak WJ, Petzold ON, Gimzewski JK: In situ mechanical interferometry of matrigel films. Langmuir 2009, 25(1):36-39.

[2] Aisenbrey EA, Murphy WL: Synthetic alternatives to Matrigel. Nat Rev Mater 2020, 5(7):539-551.

[3] Wang, X. et al. Hair Follicle and Sebaceous Gland De Novo Regeneration With Cultured Epidermal Stem Cells and Skin-Derived Precursors. Stem Cells Transl Med 5, 1695-1706, doi:10.5966/sctm.2015-0397 (2016).

**Reviewer #2-4.** Hair follicles: this point is a major content, can the authors use semi-quantitative or quantitative method to compare the differences among the fririn hydrogels and other groups? This will be important for making conclusion.

We appreciate the reviewer’s rigorous comment. We have used quantitative method to compare the differences among the fibrin hydrogels and Matrigel groups (Figure 4), which has also been added in the revised manuscript. The quantitative results showed that the average number of hair shafts increased as the wound area increased fibrin hydrogel group, with the same trend observed in the Matrigel group. Fibrin hydrogels promoted slightly greater hair follicle growth than observed for in the Matrigel group without a significant difference (N≥3).

**Page 17, lines 330–335:** *“After four weeks, the newly regenerated hair-inclusive skin tissues in all wounds were imaged under a dissecting microscope, and the relevant hair shafts were counted. The average number of hair shafts increased as the wound area increased in the fibrin hydrogel group, with the same trend observed in the Matrigel group. Fibrin hydrogels marginally promoted more HF growth relative to that observed for in the Matrigel group, without a significant difference (Figure 4A).”*

Figure 4 The numbers of hair shafts per wound in each wound and each group.

**Reviewer #2-5.** Conclusion: the concolusion section is lacking anf should be added.

Thank you for pointing this out. We have added concolusion in the revised manuscript.

**Page 24, lines 456–464** *“Whether fibrin hydrogels could be applied for HF neogenesis was determined through in vitro experiments and in vivo HF reconstruction experiments. We demonstrated that SKPs in fibrin hydrogels have high cell viability and proliferation, their stemness could be maintained, and the expression of hair induction signature genes, such as akp2 and nestin, was enhanced. Moreover, HF reconstruction experiments showed de novo hair genesis in mice, and components such as sebaceous glands and blood vessels were also regenerated. Interestingly, the regenerated hairs could persist for a long time without teratoma formation. With further advances, the fibrin hydrogels with Epi-SCs and SKPs comprise a potential therapeutic approach for alopecia and wound healing.”*

**Reviewer #2-6.** Others: Fig 1, the caption E is lacking. *, **, p value is lacking.

Thank you for pointing this out. We have added the caption E in figure legends of figure 1. *, **, p value have been added in statistical analysis and revised figures.

**Page 12, lines 275–276:** *“(D, E) SEM images of different concentration fibrin hydrogels. Scale bar: 300 μm (D), 100 μm (E).”*

**Page 11, lines 245–248:** *“All experiments were repeated at least three times, and the results were expressed as mean ± SEM unless stated otherwise. The groups were statistically compared using Student’s t-test and the statistical signiﬁcance was indicated in each bar, ns: not significant, *: P < 0.05, **: P < 0.01, ***: P < 0.001.”*

Review Editor

Comments to the Author:

Please notice that the GA should be prepared and displayed at the actual size of 1.4 inches (height) × 3.3 inches (width) or 3.6 cm × 8.4 cm following the Abstract in the submitted manuscript file.

Minimum resolutions are 300 d.p.i. for colour or tone images, and 600 d.p.i. for line drawings. The preferred font type of the texts in the GA is Arial 8-16 points (smaller will not be legible). Please do not enlarge the GA, because the image will eventually appear on the first page of the published PDF file in the specified size above. For examples of graphical abstracts, please refer to the PDF files of the published papers since 2022.

Thank you for comments. We have revised according to your comments, and we hope the revised manuscripts could meet the requirements for publication.
